# Preliminary Evaluation of a Regional Atmospheric Chemical Data Assimilation System for Environmental Surveillance

**DOI:** 10.3390/ijerph111212795

**Published:** 2014-12-11

**Authors:** Pius Lee, Yang Liu

**Affiliations:** 1 Air Resources Laboratory, Office of Oceanic and Atmospheric Research, National Oceanic and Atmospheric Administration, College Park, MD 20740, USA; 2 Department of Environmental Health, Rollins School of Public Health, Emory University, Atlanta, GA 30322, USA; E-Mail: yang.liu@emory.edu

**Keywords:** chemical analysis, ozone, atmospheric aerosols, environmental surveillance, public health tracking

## Abstract

We report the progress of an ongoing effort by the Air Resources Laboratory, NOAA to build a prototype regional Chemical Analysis System (ARLCAS). The ARLCAS focuses on providing long-term analysis of the three dimensional (3D) air-pollutant concentration fields over the continental U.S. It leverages expertise from the NASA Earth Science Division-sponsored Air Quality Applied Science Team (AQAST) for the state-of-science knowledge in atmospheric and data assimilation sciences. The ARLCAS complies with national operational center requirement protocols and aims to have the modeling system to be maintained by a national center. Meteorology and chemistry observations consist of land-, air- and space-based observed and quality-assured data. We develop modularized testing to investigate the efficacies of the various components of the ARLCAS. The sensitivity testing of data assimilation schemes showed that with the increment of additional observational data sets, the accuracy of the analysis chemical fields also increased incrementally in varying margins. The benefit is especially noted for additional data sets based on a different platform and/or a different retrieval algorithm. We also described a plan to apply the analysis chemical fields in environmental surveillance at the Centers for Disease Control and Prevention.

## 1. Introduction

Numerous epidemiological studies worldwide have demonstrated strong associations between exposure to ambient air pollutants such as ozone (O_3_) and fine particulate matter (PM_2.5_, airborne particles with an aerodynamic diameter less than 2.5 micrometer) and adverse health outcomes such as lung cancer, cardiovascular and respiratory diseases, and premature death (e.g., Jerrett *et al.* [1]; Levy *et al.* [2]; Pope and Dockery [3]). Accurate knowledge on air pollutant concentrations constitutes valuable information to assist policymakers to make informed decisions on human health. Especially useful are long-term and high-resolution air pollution forecasts or analyses at ground level where people live and breathe. In tangible terms on spatiotemporal scales, they are decadal in time and several kilometers in horizontal grid resolution. Such information based on consistency in observation protocols and model science can be used to derive epidemiological statistical outcomes. The novelty and advantage of integrating high quality observations with state-of-science physically based model to reconstruct atmospheric reality has been recognized and proven in many field studies and episodic experiments. However, only a few national institutions in the world perform routinely such chemical analysis to reconstruct and disseminate such best estimates of the air pollutant concentrations. Examples of these operational systems include CDC’s National Environmental Public Health Tracking program (http://ephtracking.cdc.gov/), and NOAA’s Air Quality Forecast service (http://www.nws.noaa.gov/ost/air_quality/). Although the demand for consistently generated and fine spatial resolution analysis atmospheric pollutant fields is acknowledged, there exist major mechanistic challenges with regard to the measurement accuracy and mathematical challenges concerning the analysis modeling systems. These challenges are particularly true for health applications. Epidemiology studies for air pollution-related diseases have stringent requirements to assure statistically meaningful and seasonally and regionally representative data sets used to devise study plans and to derive hypotheses. 

This study consists of the first few steps toward: (i) building a prototype regional chemical analysis system at the National Oceanic and Atmospheric Administration (NOAA) Air Resources Laboratory (ARL) and (ii) positioning it as an operational service reliably maintained by a national center for air pollution scientists, air resources managers and the general public. In order to fulfill NOAA’s mandate of sustaining vibrant economic growth despite the potential adverse effects of air pollution episodes [4], the system must have a broad range of end users who utilize the information provided to minimize hazards and mitigate impacts. The same service will be referred to as a re-analysis product when it is generated by a subsequent re-run of the modeling system updated with improvements beyond the initial analysis due to upgrades in data retrieval algorithms and/or advances in the physics-based models.

Section 2 describes the ARL regional chemical analysis system (termed ARLCAS hereafter). The ARLCAS is being built with much interest and input from experts across the academia and governmental agencies. In particular, NASA’s Applied Science Program has funded a five-year Air Quality Applied Science Team (AQAST) project since 2011 and has recently included building a prototype of the ARLCAS under the auspices of one of its “quick turnaround” Tiger Team projects [5]. This project started in June 2014. The long-term commitment of the ARLCAS at NOAA prompted us to report its progress and this potentially major service to the human health scientist community (e.g., Janes *et al.*, [6]).

Section 3 demonstrates the concept and success of integrating the Aerosol Optical Depth (AOD) data observed by the Moderate-resolution Imaging Spectro-radiometer (MODIS) to constrain the surface concentration of particulate matter less than 2.5 μm diameter (PM_2.5_) by using a variant of the ARLCAS. This slight digression in using a variant of the ARLCAS is necessary to expedite testing and investigation of the various observation data sets and assimilation schemes. Besides assimilation of the MODIS AOD, we also assimilated the U.S. Environmental Protection Agency (EPA) AIRNow network surface air pollutant measurements with two different assimilation temporal frequencies. These tests serve to quantify the incremental improvements when additional data sets are assimilated to constrain the ARLCAS. Section 4 discusses how the ARLCAS outputs can benefit its potential user: CDC’s National Public Health Tracking program. In particular, this section focuses on the practicality of such applications. Finally, Section 5 is the conclusion of our study.

## 2. Building ARLCAS

**Figure 1:**
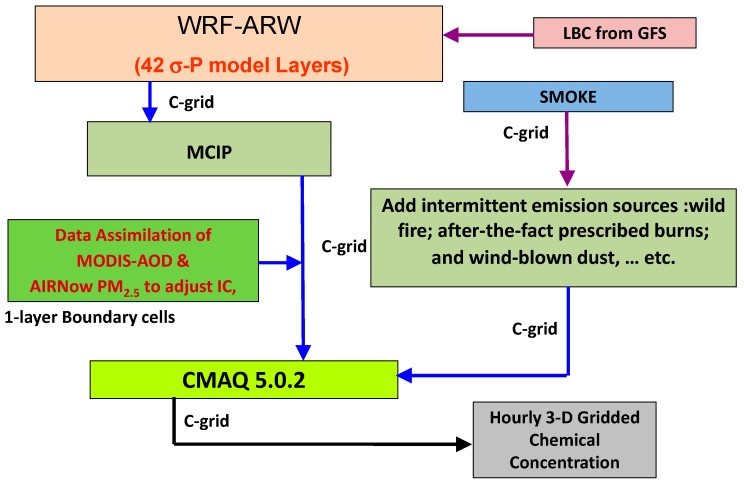
Schematic diagram of the ARLCAS.

We set out to build the ARLCAS to provide air-quality analysis fields over the continental U.S. (CONUS). The immediate focus is on providing a long-term record analysis field for surface PM_2.5_ in a spatial and temporal resolution over CONUS that is acceptable to epidemiologists specialized in air pollution-related diseases. Ideally, there should be a reasonable degree of homogeneity in the records in terms of the continuity of observation data, retrieval algorithms, emission inventory and chemical transport model (CTM) parameterization packages. Figure 1 shows a schematic summary of the ARLCAS being built and tested.

The left hand side column of Figure 1 represents the four major sequential processing in the ARLCAS. Starting with the Weather Research and Forecasting (WRF) numerical weather prediction model with the Advance Research WRF dynamic core (ARW) the system aims to reconstruct the meteorology [7]. WRF-ARW in the ARLCAS utilizes large groups of observed meteorological data to constrain its physically-based meteorological simulation. The top box on the left hand side column represents that the WRF simulation is fed by the NCEP global forecasting system (GFS) forecast derived lateral boundary conditions (LBC). The modeling system also takes advantage of the meteorological observation taken inside the CONUS. Subsection 2.1 expands on this.

The second box on the left hand side column labeled as MCIP, is a met-driver to chemical modeling interface processor. Immediately underneath it, lies a data assimilation module, the center-piece of the ARLCAS system that ingest observation data at various intervals with a state-of-science data assimilation algorithm. At each of these ingests the concentration fields are updated to reflect the adjustment constraint by the observation ingested. The bottom box in the left hand side column labeled as CMAQ 5.0.2 depicts the name and version of the forward model used; namely, the U.S. EPA Community Multiple-scale Air Quality model version 5.0.2 [8]. 

The two boxes in the middle of the right hand side column in Figure 1 are the modules that generate CMAQ-ready emission by an inventory based estimation handled in the U.S. EPA Sparse Matrix Operator Kernel Emission (SMOKE) and by near real-time intermittent emissions that can be remotely sensed as shown in a separate box underneath SMOKE in Figure 1. The bottom right box represents the 3D analysis fields produced by a short execution of the CMAQ model. As output from CMAQ, they are assured of consistencies in chemical activity, temporal and spatial gradients. Furthermore, by short simulations not to exceed 6 h, the output remains strongly constrained by the ingested observations. 

### 2.1. WRF Meteorological Model Physics Package

One important advantage of the ARL system is its close tie with the U.S. National Centers for Environmental Prediction (NCEP) North American Mesoscale (NAM) modeling system in terms of sharing NAM’s initialization files and its dynamic constraints from boundary conditions provided by the NCEP Global Forecasting System (GFS). These constraints are derived by three major observation data assimilation systems: (1) the GFS Data Assimilation System (GDAS) [9], (2) the NAM Data Assimilation System (NDAS) [10] and (3) the land surface model (LSM) Data Assimilation System (GLDAS) [11]. These meteorology assimilation systems have operational status in the NCEP national center and each complies with a common 3D Variational (3DVar) protocol called the “Grid Point Statistical Interpolation” (GSI) (e.g., Wu *et al.*, [12]). GDAS, NDAS and GLDAS are critical to assure the best possible initializations and boundary conditions for WRF-ARW within the ARLCAS setup to predict accurately the dynamical and thermal states of the atmosphere. They utilize broadly comprehensive and vigorously quantity-assured observations from land-, air- and satellite-based platforms across the globe.

**Figure 2:**
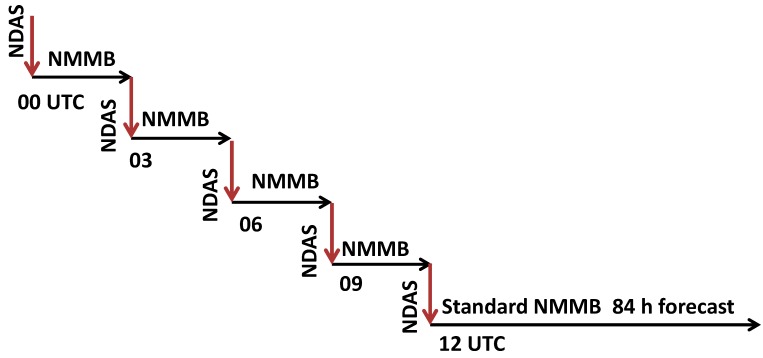
Schematic diagram of four sequences of the NDAS and NMMB short forecasts to prepare initialization condition for a standard 12 UTC cycle NAM forecast.

Both the GDAS and NDAS are run at NCEP with four daily cycles initialized at 00, 06, 12 and 18 UTC. The GLDAS is run daily. Although the exact sampling pool of observational data derived may vary over time due to instrumentation retirement or failure and/or maintenance, they all utilize vastly diversified and large sets of quality-assured observations. The GLDAS model is run first, followed by GDAS and then NDAS. Each of these three data assimilation systems is run alternating with its corresponding forward model. The GDAS, GLDAS and NDAS have the following corresponding forecast models: NCEP’s GFS [13], the NCEP Oregon State University, Air Force and Hydrologic Research Laboratory (NOAH) Land Surface Model (LSM) [14] and NCEP’s NAM with Non-hydrostatic Mesoscale Model with Arakawa B grid staggering (NMMB) [15]. The GLDAS and GDAS are the first models to be executed in the modeling system, as the other NCEP operational models depend on them directly or indirectly. Besides this dependence, the GDAS and NDAS data assimilation systems are conducted similarly in terms of their data assimilation and forward model-interplay sequencing (Figure 2).

We take the daily 12 UTC forecast cycle of NAM (*i.e.*, the NCEP NDAS data assimilation and NMMB forward model pair) to illustrate their sequencing interplay. This process has the following steps: (a) perform the NDAS step to derive the best guessed field, *a posteriori*, based on the NCEP GSI protocol started 12 h prior (*i.e.*, initialized at 00 UTC of the same forecast day) using the most recent GFS forecast output over the NAM as a priori; (b) conduct a 3 h NMMB forecast initialized from the best guessed field that resulted from step (a) and (c), carry out steps (a) and (b) sequentially to completion. This completes the initial paired sequence of data ingest and short forecast. In the next stage, we (d) repeat step (c) three more times sequentially at 03, 06, 09 UTC (but each uses the previous short NAM forecast output at the end of the third forecast hour as *a priori* and accounts for all “on-time” reported and quality-assured observations). Finally, (e) launch a standard NAM forecast for the 12 UTC cycle. This is optimally initialized by the completion of step (d) with the maximum amount of observations reported during the 12 h prior. The free forecast in step (e) is only constrained by the boundary conditions derived from the GFS. Figure 2 shows a schematic of the linkage among these four sequences of NDAS and NMMB short forecasts in preparation for the initial conditions for a standard forecast by the NAM.

In the ARLCAS for CONUS, the initialization and boundary conditions obtained in step (e) is used. The sea surface temperature (SST) is updated daily by NCEP real-time global SST analysis in 0.5 degree grid spacing [16].

The WRF-ARW meteorological model with dynamic core version 3.4.1 [7] is used as the meteorological driver in ARLCAS. One of the two overarching factors supporting this selection is its good compatibility in some of the physics packages between WRF and the U.S. EPA Community Air Quality Multi-scale Model (CMAQ), the selected regional CTM in ARLCAS. The other rationale is the common Lambert Conformal Conic map projection for WRF-ARW and CMAQ. With a common map projection and perhaps the deliberate design of the vertical grid spacing, one can devise identical grid structures in both the horizontal and the vertical grids for the meteorological model as well as the CTM used, thus removing the necessity to carry out a variable transform due to grid differences. Otherwise, this necessity could arise in the coupling of the meteorological model and the CTM regardless of whether an off-line or in-line approach is used. In the ARLCAS, the off-line approach is taken.

### 2.2. Meteorological Data Ingested by the NOAA NCEP National Center for Weather Prediction

The observation data received by NCEP are vigorously quality-assured with respect to various criteria in accordance with the specific instrumentation characteristics and acquisition algorithms. The interested reader is encouraged to read about the vast amount and types of land-, air- and satellite-based meteorological observation data GDAS, NDAS and GLDAS acquire, quality-assure and ingest (e.g., [17,18]). For the sake of completeness, the major multiple daily meteorological observation data used for the initialization of the ARLCAS via the NAM system handled by the assimilations are briefly described and organized below in order of surface-, air- and space-based platforms. NCEP has access to many land-, air-, and space-based observations that report multiple times daily in near real-time. There is land-based equipment (at least 5000 automated rain gauges, 150 Weather Surveillance Radar 88 Doppler (WSR-88D) radars for wind velocity and rain reflectivity); as well as observation networks comprising over 1800 meteorological stations in airports and surface synoptic observation station networks that report basic thermal and dynamic variables such as temperature, dew point depression, cloud cover, visibility, barometric pressure, and wind direction and velocities within CONUS. A sizable network exists comprising a few tens of Radio Acoustic Sounding Systems that reports planetary boundary layer (PBL) structure, temperature and wind profiles. Near real-time precipitation reports from the NOAA River Forecast Centers and Hydro-meteorological Automated Data System (HADS) and SNOwpack TElemetry (SNOTEL) data set and from the integrated Flood Observing and Warning System (FLOWS) precipitation data are also transmitted to NCEP. A vast MESONET network comprises over 5000 measurement stations that tailor measurements to capture mesoscale phenomena such as the advance of a squall line spread extensive in CONUS. MESONET consists of multiple networks for various primary purposes, such as the Co-operation Observer Program (COOP), Co-operative Research Network (CRN), Office of HYDROlogy, NOAA (HYDRO) and SNOW networks [17].

Along the coasts and within the inland water bodies of CONUS, several hundred participating buoys and ships report temperature, wind and surface pressure. About 60 marine-based observation stations from ships, buoys, tidal gauges and the Coastal-Marine Automated Network (C_MAN) report on the surface air conditions at the sea surface level. C_MAN operates in concert with other oceanographic data such as temperature and the salinity profile of the coastal waters from buoys applicable to GDAS. Aircraft Reports (AIREP) from automated weather stations from airports around the world also serve GDAS.

The cost effectiveness of meteorological observation instrumentations varies widely. For instance, the NOAA’s P-3 aircraft Hurricane Hunter is deployed to measure sea surface wind speed during a hurricane using a stepped frequency microwave radiometer. It also discharges dropwindsondes, each of which contains an expendable weather reconnaissance device and a global positioning system (GPS)-enabled location tracking device, that descend to the surface under a moderate terminal speed slowed down by a parachute. An averaged descent takes 3 to 5 minutes during which multiple readings on spatial coordinates, wind speed and direction, barometric pressure, temperature and humidity are measured and relayed back electromagnetically to a central receiver. These readings are highly valuable but costly to acquire. On the other hand, aircraft-based instrumentations from many major commercial airlines are much less expensive. Real-time reports are relayed back via radio transition. They report air temperature, wind speed and wind direction along flight paths using the Aircraft Communications Addressing and Reporting System (ACARS), the Meteorological Data Collection and Reporting System (MCDRS), the Aircraft Meteorological Data Reports and the Tropospheric AMDAR (TAMDAR) [18] reporting standards. Over CONUS, there are roughly 300 three-minute interval reports on relatively humidity and approximately 7000 three-minute interval reports on temperature, wind speed and direction. Furthermore, Pilot balloons (PIBAL) with optical theodolite for position measurement are launched twice daily at 00 and 12 UTC to measure upper air wind direction and wind speed. In the free troposphere, there are about 1100 routine *in situ* measurement synoptic RAdiosonde/Rawinsonde Observations (RAOBS) over CONUS with launches at 00 and 12 UTC.

### 2.3. Assimilation of Remotely Sensed Data from Various Environmental Satellites

Remote sensing from satellite-based instrumentation has revolutionized meteorological data acquisition. In accordance with a recent efficacy comparison (e.g., Crichton *et al.*, [19]) between conventional technology and satellite-based technology, it was concluded that they were about equally effective. It is possible that the continual trend is that remote sensing will stay comparable with conventional observation approaches.

On board NOAA Geostationary Operational Environmental Satellites (GOES) 15–19, a special Sensor Microwave Imager Sounder (SSM/IS) measures emissivity data and Time Domain Reflectometry (rain-free TDR) measures soil moisture products for surface and root-zone soil. In addition, ATOVS (Advanced TIROS (Television and Infrared Observational Satellite) Operational Vertical Sounder) and the High Resolution Infrared Radiation Sounder (HIRS/4) instrument measure the incident solar radiation primarily in the infrared frequencies of the spectrum via 19 channels.

The Atmospheric Infrared Sounder (AIRS), an advanced sounder containing 2378 infrared channels and four visible/near-infrared channels, is an important data provider. It aims to obtain highly accurate temperature profiles within the atmosphere. It also generates and relays Earth/atmosphere products to data retrieval centers. AIRS features instruments in the AIRS/AMSU-A/HSB triplet, which together accurately measure temperature and humidity profiles in the atmosphere.

The Infrared Atmospheric Sounding Interferometer (IASI) is precisely calibrated. It measures temperature and water vapor profiles as well as numerous atmospheric species. It is specialized to measure long-lived climate gases such as carbon dioxide (CO_2_), methane (CH_4_), O_3_ and carbon monoxide (CO). Moreover, the Advanced Very High Resolution Radiometer (AVHRR) is a radiation-detection imager that can be used for remotely determining cloud cover and the surface temperature.

The Suomi National Polar-orbiting Partnership (NPP) is the newest of the American Polar Orbiting Environmental Satellites (POES). On board, there is the Advanced Technology Microwave Sounder—low-atmosphere moisture measurements (ATMS); the Visible Infrared Imaging Radiaometer Suite (VIIRS) that provides critical data for monitoring global weather and climate such as cloud properties; and the Cross track infrared sounder (CrIS) that measures temperature, pressure and moisture profiles. Through those measurements, it is anticipated that the science community will understand the El Nino and La Nina phenomena better; further, the Ozone Mapping Profiler Suite (OMPS) extends the over 25-year total ozone and ozone profile records. These records are used by ozone assessment researchers and policymakers to track the health of the planet’s ozone layer. The improved vertical resolution of OMPS data products allows for better testing and monitoring of the complex chemistry involved in ozone destruction near the tropopause. In addition, the Clouds and the Earth's Radiant Energy System (CERES), a three-channel radiometer, measures both solar-reflected and Earth-emitted radiation from the top of the atmosphere to the surface. It also determines cloud properties including the amount, height, thickness, particle size and phase of clouds using simultaneous measurements by other instruments. 

Common to all these platforms is that they can all use water vapor detection channels to derive wind speed. Geostationary satellite-derived atmospheric motion vectors (AMVs) are generated by incorporating GOES and POES imagers and forecast data from a numerical model. The principle of wind derivation is used to follow a recognizable tracer (cloud and water vapor features in infrared windows and water vapor bands) in a sequence of images, and derive its apparent velocity. The Scatterometer on board these GOES and POES satellites uses microwave frequencies for measuring wind as well as air–sea interaction, while climate studies are particularly useful for monitoring hurricanes.

The Tropical Rainfall Measuring Mission (TRMM) satellite measures rainfall with heightened sensitivity in the tropics. On board TRMM, there is the Precipitation Radar (PR), TRMM Microwave Imager (TMI), Visible Infrared Scanner (VIRS), CERES and Lightning Imaging Sensor (LSI). The TMI is the main instrument used for precipitation. It tends to underestimate precipitation at higher latitudes. Its precipitation radar algorithm likely underestimates precipitation in regions of intense convection over land. Its merged product released in 3B43 (http://trmm.gsfc.nasa.gov/3b43.html) has issues that hamper its application to climate studies.

Naval Oceanographic Office (NOO) and National Environmental Satellite Data and Information Services (NESDIS) are reliable agencies to obtain remotely sensed SST measurements, daily snow/ice analysis as well as an ozone, aerosol, vegetation fraction and climatology leave area index (LAI). All these remotely sensed data are critical to the success of ARLCAS.

### 2.4. WRF Configuration and Physical Features Applied

The WRF-ARW meteorological model version 3.4.1 was used in this initial version of the ARLCAS. Both the WRF and CMAQ model domains have 12 km horizontal grid spacing. There are 42 unevenly spaced vertical sigma layers extending the surface to 100 hPa, with higher resolution near the ground and at the PBL top and free troposphere interface to vigorously capture phenomena near the surface as well as the heights where PBL venting occurs, respectively [20]. The first full sigma level is about 8 m above ground level. WRF-ARW and CMAQ are coupled in an off-line fashion.

At the outset, we decided to match the physics package of WRF and CMAQ as much as possible to attain a high degree of consistency between these two major components. In particular, we hope to assure good consistency between the physics parameterization schemes to model the PBL. WRF’s major science packages chosen for the ARLCAS are summarized in Table 1.

**Table 1:** Physics packages configured for the WRF-ARW component of ARLCAS.

Phenomenon Addressed	Parameterization Scheme	Remarks and Reference(s)
Advection	Runge-Kutta 3 advection scheme	Wicker and Skamarock [21]
Short wave and long wave radiation	Rapid Radiative Transfer—Goddard	Lacono *et al.* [22]
PBL turbulent mixing	Asymmetric Convective model2	Pleim [23]
Cloud convection mixing	Betts-Miller-Janjic mass adjustment	Ping and Luo [24]
Surface layer heat/momentum exchange	Monin-Obukhov similarity theory	Monin and Obukhov [25]
Land surface exchange	NCEP NOAH land surface model	Ek *et al.* [14]

The WRF-ARW output is processed by the Meteorology-Chemistry Interface Processor (MCIP) (Otte and Pleim, 2010 [26]) to window out fields for the CMAQ domain, to distribute static pollutant emission flux rates and to diagnose a few proprietary parameters for CMAQ. A recent MCIP release version 4.2 is used. MCIP receives a meteorological data feed from WRF hourly and generates an hourly output to feed CMAQ. It also reads emission inventory files such as from point and area sources and distributes them as time-varying flux rates at hourly intervals in accordance with the day of the week, holidays and in correspondence with seasonal and locational influences. MCIP also does diagnostic computation for depositional speeds and for the plume rise of buoyant pollutant plumes. Basically, the emission processing approach taken follows those performed for that in the National Air Quality Forecasting Capability (NAQFC) (Pan *et al.*, [27]). 

### 2.5. Area Sources

We used the U.S. EPA projected 2012cs non-road area sources directly and we merged it with U.S. EPA 2012aa version of the National Emission Inventory (NEI) for other sectors in the area source category. The ARLCAS CONUS domain involves emissions from our neighbors (see Figure 1). We merged in the Environment Canada-provided Canada 2006 Emission Inventory for Canada and the National Emission Inventory Mexico 1999 for Mexico. 

### 2.6. Mobile Sources (On-Road)

We used both the U.S. EPA 2005 and 2012 NEI on mobile emissions to derive a basis for temporal scaling emission fluxes from mobile emissions over specific grid points. We followed the Cross-State Air Pollution Rule (CSAPR) NEI for those two years as the basis for scaling sources within CONUS to the year of interest. This approach stemmed from our experience accumulated through the past decade ARL monitored the performance of NAQFC [27]. It is believed that the NAQFC treatment of mobile sources contributes significantly to the reduction of the over-prediction of surface O_3_ concentration (Stajner *et al.*, [28]). Its methodology is repeated here. The NEI accounts for the time-activity pattern, week/weekend and diurnal variability for different vehicle types. This is not ideal given that it would miss the emission fluctuation due to the economic downturn between 2008 and 2010. A comparison of projected emissions was performed between surface and satellite observations (Tong *et al.*, [29]). This is a compromise between a recently updated data set and a contiguous time span between base years to provide a statistically significant trend.

### 2.7. Point Sources Energy Generation Units (EGUs) and Non-EGUs

We used the U.S. EPA 2005 NEI as a base year. We superimposed on it updates from the latest available quality-assured readings from the Continuous Emission Monitoring (CEM) network for EGUs and non-EGUs. The emission fluxes from these units were then interpolated or extrapolated to the year of interest by using the U.S. Department of Energy forecast energy consumption outlook factor. 

### 2.8. Detected Real-Time Wildfires

We chose to use a hot spot and smoke plume detection product from the NOAA Hazard Mapping System (HMS) (Ruminski *et al.*, [30], Rolfe *et al.*, [31]), which blends multiple satellite retrievals and human analyst products to provide detection and hot-spot counts of wildfires over the U.S. In this work, the HMS product is used to quantify fire emissions. The HMS detects wildfires by using a combination of seven NASA and NOAA satellites. Upon the determination of “wildfire hot spots” by the HMS, the U.S. Forest Service Bluesky Framework provides inventories for fuel type and fuel loading to determine the intensity and projected duration of these fires (Larkin *et al.*, [32]). By considering variability in fuel loading and availability, some of the fires are assumed to last for the next 6 h and thus are qualified to become emission sources for the CMAQ forecasting runs. The recommendation of “ratio to CO” by Hsu and Divita [33] to estimate NO_x_, taken as NO plus NO_2_, VOC, NH_3_ and SO_2_ emission fluxes from wildfires, is employed. From Bluesky, the heat release associated with the fires is applied to the Briggs’ equation (Briggs, [34]) to calculate injection heights for these buoyant emissions.

### 2.9. Gas-Phase Emissions due to Biogenic Sources and Lightning, and Aerosol Emissions due to Sea Spray

Terrestrial biogenic emissions are handled by either an off-line pre-preprocessor or an in-line CMAQ model embedded module based on the Biogenic Emissions Inventory System (BEIS) (Guenther *et al.*, [35]) version 3.14, which accounts for drought stress (e.g., [36]). Lightning NOx is accounted for by using an in-line emission generation module within the CMAQ model. The National Lightning Detection Network (NLDN) including information on signal strength and the multiplicity of flashes is specified in the emission rate calculations. For coarse mode sea salt particle emissions, CMAQ utilizes an ocean shoreline and surf zone mask file to provide static information to an uptake and splash zone wave breaking equation with 10 m gust wind speed as the input to generate emission fluxes.

### 2.10. Dynamic Lateral Boundary Condition (LBC)

Output from the NESDIS Real-time Air Quality Modeling System (RAQMS) global model [37] was selected to provide the basis for the derivation of the time-varying chemical LBC for ARLCAS. The LBC for ARLCAS was derived from RAQMS’s six-hourly output fields. The time-varying chemical LBCs include full-profile O_3_, CO, sulfur oxidants, nitrogen oxidants and volatile organic compounds (VOCs). Species mapping tables were used to convert the LBC species concentrations of RAQMS to CMAQ’s implementation of the CB05 gas phase chemical mechanism. RAQMS is excellent at reproducing O_3_ concentration in the upper troposphere. A statistical digital filter analysis system [38] was used in the RAQMS data assimilation to perform a univariate assimilation of the stratospheric profile and total column ozone observations from Aura Ozone Monitoring Instrument (OMI) satellite data. The RAQMS gas phase mechanism explicitly treats ethane (C_2_H_6_) and uses different lumping methods for alkenes. The RAQMS gaseous species AONE (acetone), OLET (terminal alkenes) and OLEI (internal alkenes) are primarily mapped to the CMAQ species of PAR (alkane). For aerosol species, the RAQMS species of dust particles are mapped to the CMAQ aero5 species of A25J (others) and ASOIL (crustal minerals). The RAQMS sea salt particles are mapped to the CMAQ aero5 ANAJ, ACLJ and ACLK (accumulation mode sodium, chloride and potassium). The static monthly chemical boundary condition derived from an annual run of GEOS-CHEM for 2006 after the procedure and speciation mapping of Tang *et al.* [39] provides a backup should the RAQMS data become unavailable.

### 2.11. CMAQ Physics and Chemistry Packages

Our configured CTM comprises the CMAQ model version 5.0.2. The CTM solves the continuity equations of air pollutants written as follows:

(1) $$\frac{\partial \phi_{i}}{\partial t}=-\nabla \cdot V\phi_{i}+\nabla \cdot (K_{h}\nabla \phi_{i})+P_{chem}-L_{chem}+E+{\left(\frac{\partial \phi_{i}}{\partial t}\right)}_{drydeposition}$$

where *φ_i_* is the mixing ratio of the *i*th species; *V* is the wind vector; *K_h_* is horizontal diffusivity; *P_Chem_* and *L_Chem_* are the chemical kinematic rate of production and loss for *φ_i_*, respectively; *E* represents emission fluxes; and is the last term on the right hand side is the loss rate due to dry deposition. Implicit in Equation (1) are the multiple inputs from WRF-ARW such as air and surface skin temperatures; air mass speed, direction and moisture; water condensate content; and canopy water and conductance that govern dry depositions. It is believed that these meteorological inputs are as decisive as pollutant emissions in determining the accuracy of the analysis chemical fields.

Table 2 summarizes the main components of the parameterization modules selected. We chose the Carbon Bond Gas Phase Mechanism 2005 (CB05) (Gery *et al.*, [40]; Sarwar *et al.*, [41]) coupled with the aero5 aerosol module that expresses the aerosol size distribution of the atmospheric particles in three log-normal distributions defined with their characteristic geometric diameters to account for the Aitken, accumulation and coarse modes (Binkowski and Shankar, [42]). The chemistry modules have 132 species and 156 reactions. CMAQ version 5.0 has been tested extensively in various regulatory and forecasting studies and has shown reasonably good performance (e.g., Fu *et al.*, [43]).

**Table 2:** CMAQ version 5.0.2 chemistry and physics packages.

Phenomenon Addressed	Parameterization Scheme	Remarks & Reference(s)
Advection	Piece-wise parabolic method	Mathur *et al.* [44]
PBL turbulent mixing	Asymmetric Convective model2	Pleim [23]
Cloud convection mixing	Asymmetric Convective model	Mathur *et al.* [44]
Surface layer heat/momentum exchange	Monin-Obukhov similarity theory	Monin and Obukhov [25]
Gas phase chemistry	Carbon Bond Mechanism 2005	Sarwar *et al.* [41]
Photolytic attenuation by clouds	WRF clear sky flux and cloud fraction	Mathur *et al.* [44]
Aerosol size distribution	Tri-modal log-normal distribution	Binkowski and Shankar [42]
Aerosol chemistry	Module Aero5 of CMAQ5.0.2	Binkowski and Shankar [42]
In and below cloud scavenging	Use hydrometeor fields from WRF	
Dry deposition	M3Dry	Pleim and Ran [45]

In light of the importance of the consistency of the dynamical and thermal parameterization of the physical processes, the hydrometeor growth and removal processes as well as the radiative balances and the cumulus and planetary boundary and microphysical processes are chosen to be as close as possible to those in WRF-ARW. Similarly, the convective cloud mixing scheme and planetary boundary scheme were set to the Asymmetric Convective Model schemes (Pleim, [23], Mathur *et al.*, [44]), identical to their respective ones in the WRF model. The radiation fluxes as well as the land-air interface exchange fields in CMAQ are directly received from MCIP as a prognostic input from WRF. 

## 3. Chemical Analysis Testing

The methodology of using air chemistry and its related observations to constrain air quality models is sometimes called chemical data assimilation (e.g., Carmichael *et al.*, [46], Elbern *et al.*, [47], Sandu and Chai, [48]). In this initial effort, we assimilated MODIS column-integrated AOD attributable to both fine and coarse mode atmospheric aerosols and the surface measurement of PM_2.5_ compiled by the U.S. EPA AIRNow surface network. Specific assimilation techniques are required for both these data sets: (a) MODIS AOD Collection 5 obtained from the Aqua and Terra satellite platforms are column-integrated extinction properties of atmospheric aerosols. The redistribution of the influence of this two dimensional (2D) value to the 3D particle size and chemical species-specific aerosol fields is an active research area. (b) The AIRNow network reported surface PM_2.5_ concentration hourly. This has about 400 active monitoring stations at any given area rather being unevenly distributed across CONUS (more densely deployed in the Northeast than in the Western parts of the country). Fortunately, they are rich in content considering the frequencies and geographical coverage of these sets. In assimilating these observations (obs), an Optimal Interpolation (OI) algorithm is adopted. One can interpret OI as a simplified extended Kalman Filter formulation in terms of setting an influence distance beyond which observations exerts no influence on that spatial point of interest. For instance, the distance for an AIRNow observation to have influence on the centroid point of a 12 km CMAQ grid is about 200 km [48]. At each instance when OI is invoked, the following analysis problem is solved:

(2) $$X^{a}=X^{b}+BH^{T}{\left(HBH^{T}+O\right)}^{-1}(Y-HX)$$

where *X* and *Y* are the state and observation vectors, respectively. *B* and *O* are the background and observation error covariance matrices. *H* is the observational operator. The superscripts *a* and *b* indicate analysis and background states. Observations far beyond the calculated background error correlation horizontal length scale thresholds would have no effect in the analysis.

### 3.1. Assimilation of MODIS AOD

In the current study, daily data injection takes place at 17 UTC. The time spans the overlap between the descending node and ascending node passing over the Equator by Terra and Aqua is 10:30 a.m. and 1:30 p.m. local time, respectively. We chose to ingest the assimilated information derived from the AOD measurements from both satellites through an initialization adjustment injected into the *priori* given by the 5th h forecast result of the previous 12 UTC forecast cycle. The so-called NOAA National Meteorological Center (NMC became NCEP in 1995) method is used. In the NMC method, the error covariance matrix is constructed by using multiple forecasts for the same grid cell and same temporal stamp.

### 3.2. Assimilation of AIRNow PM_2.5_

The Hollingsworth–Lönnberg observational method for deriving the background error covariance matrix is more attractive for this data set (Hollingsworth and Lönnberg, [49]). This method provides a reliable estimate of the background error variance as well as the ratios between the observation error variance and background error variance (Chai *et al.*, [50]). These values determine the weightings between the two terms *B* and *O* in Equation (2).

### 3.3. Test Results of the OI Data Assimilation Scheme

During the development of the ARLCAS, it is necessary to test the efficacy of the OI scheme. Unfortunately, ARLCAS has not been built to the stage where we can reliably test the OI component over an extended period of time. We performed two variants of the system to expedite quantification on the accuracy of the analysis field compared to that by a free forecast. The former was a limited area study and the latter an investigation of incremental improvement by gradually expanding the observation sets.

**Figure 3:**
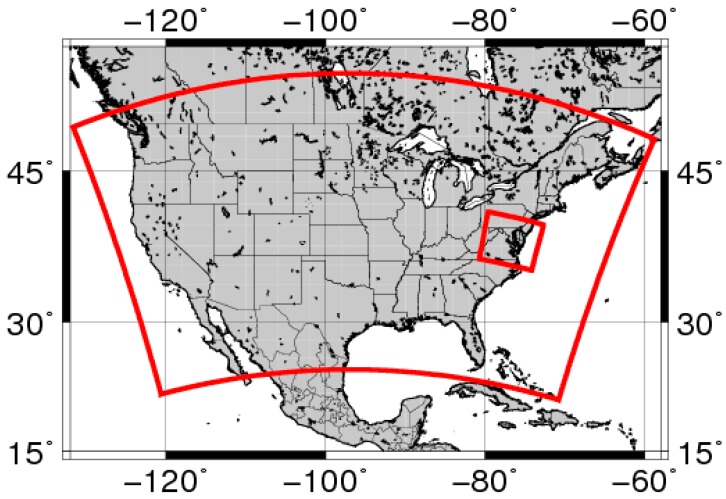
Domain of the ARLCAS regional system covers the continental U.S. (outer red frame). The inset (inner red frame) shows the domain used in a mimicked state implementation plan simulation over MD in Section 3.

The limited area study utilized ARLCAS in a smaller geographical coverage within CONUS which is substantial larger than the Eastern U.S. We used the analysis field thus generated to provide initialization condition (IC) and lateral boundary condition (BC) fields for a mimicked State Implementation Planning (SIP) simulation for the State of Maryland (see inset in Figure 3). The SIP modeling by the Maryland Department of the Environment utilized an offline WRF-CMAQ system much resembles that described in Table 1 and Table 2. Two SIP modeling sensitivity simulations were run using ARLCAS output to derive IC and BC between July 1 and 10 2011. The SIP simulation WRF-CMAQ system was restarted every 24 hours initialized at 12 UTC with: (SIP) no data assimilation, and (SIP5) with ingest of total AOD retrievals for Terra and Aqua at 17 UTC and 1 h forward averaged AIRNow PM_2.5_ injected every 6 h. The runs were evaluated by measurements from a subset of the AIRNow measurements within the SIP model domain totaled 36 PM_2.5_ stations. Table 3 illustrates the metrics used to rank the performance of the runs for surface hourly PM_2.5_ concentration. It was shown that the SIP5 Case improved the performance of the mimicked SIP simulation. It reduced the Root Mean Square Error (RMSE) significantly, by a factor of 4.2. 

**Table 3:** Statistical metrics based on 36 hourly reporting PM_2.5_ monitors for Base and SIP5 cases.

Case	Observed Mean (µg∙m^−3^)	Mean Bias	RMSE	Correlation Coefficient
SIP	19.8	−0.67	24.28	0.49
SIP5	19.8	1.15	5.79	0.53

**Figure 4:**
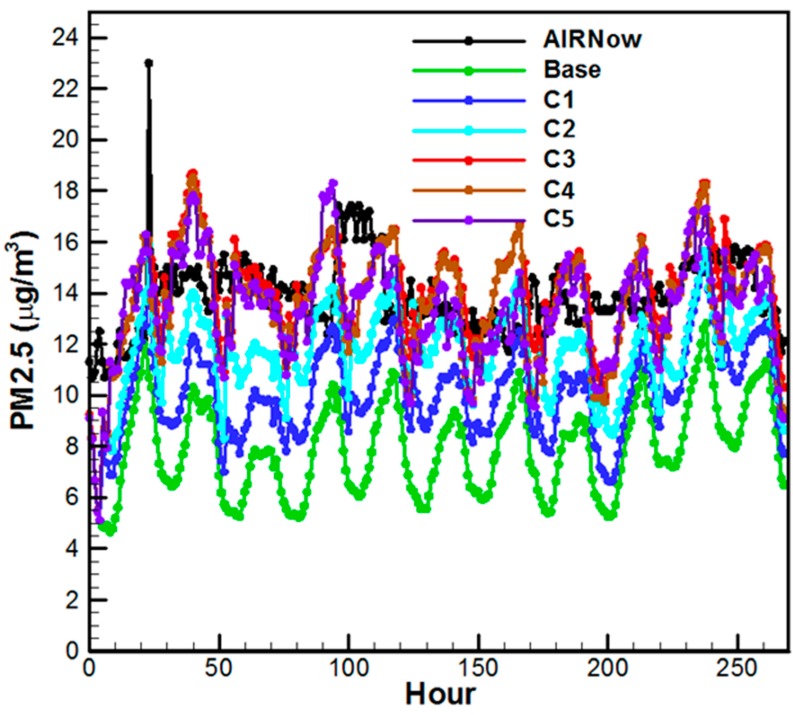
Comparison of domain-wise hourly surface PM_2.5_ concentration with measured values from AIRNow stations—averaging 1100 reporting stations over CONUS for each hour. For the Base Case there is no data assimilation. The other cases invoked OI assimilation with incremental addition of obs: (C1) fine mode AOD from Terra, (C2) add to the previous Case coarse mode AOD from Terra, (C3) add Total AOD from Aqua, (C4) add AIRNow PM_2.5_ measurements reported at 12 UTC and invoke OI at 12 UTC, and (C5) add to C3 AIRNow measurements every 6 h.

We also resorted to a variant of the system that consists of the NMMB dynamic core of the WRF model whose retrospective output we have proprietary access as well as to a slightly older version of CMAQ. This digression accelerates our development of the OI algorithm of ARLCAS since the long-term performance of the OI scheme is critical and testing it over extended periods populates meaningful statistics. We chose July 1 through 14 2011 for our test period. A base run (Base) with no OI of chemical data and five sensitive runs were performed with OI data assimilation operated on observations with various degrees of data comprehensiveness and algorithm sophistication (see Figure 4). Description of the five sensitivity simulations with incremental increases in chemical observations and OI assimilation frequencies is as follows. It accounts for obs: (C1) fine mode AOD from Terra, (C2) add to the previous coarse mode AOD from Terra, (C3) add Total AOD from Aqua, (C4) add AIRNow PM_2.5_ measurements reported at 12 UTC and invoke OI at 12 UTC, and (C5) add to C3 AIRNow measurements every 6 hours. Figure 4 shows the run results for CONUS domain-wise hourly averaged surface PM_2.5_ verified with AIRNow hourly measurements. Incremental improvement is realized. It is shown that with the increment the PM_2.5_ concentration in the analysis field better matches the measurements.

## 4. Potential Value for Environmental Surveillance

The ARLCAS is designed to support environmental surveillance through the CDC Environmental Public Health Tracking Network (EPHTN). The EPHTN is a dynamic web-based system (http://ephtracking.cdc.gov) that tracks and reports environmental hazards and the related health endpoints (McGeehin *et al.* [51]). Established in 2002, this program addresses the critical information gap outlined by the Pew Environmental Health Commission report in 2001 that the environmental public health system was fragmented, neglected, ineffective and unable to adequately respond to environmental threats. The Pew report recommended a coordinated public health system to integrate information on environmentally related diseases, human exposures and environmental hazards to better respond to and reduce environmental health threats. With these requirements in mind, the EPHTN was designed to disseminate information to guide policy, practice and other actions to improve the nation’s health, and advance environmental public health science and research (CDC [52]).

**Figure 5:**
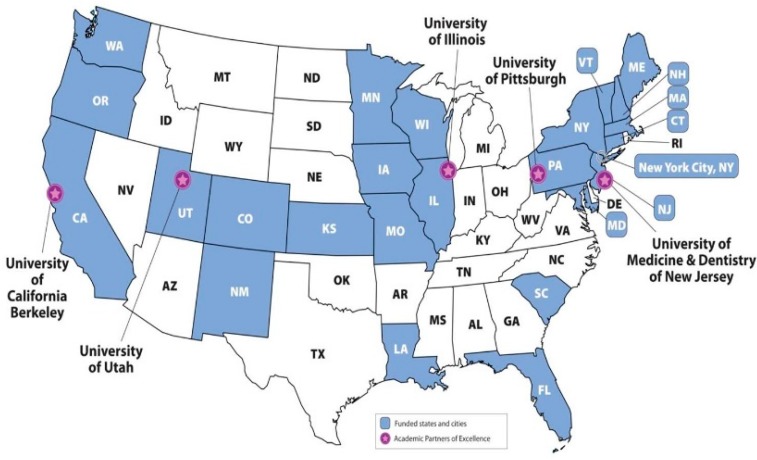
Current tracking states and academic partners.

By 2010, 23 states and New York City had joined the EPHTN (Figure 5). Currently, Tracking provides 34 nationally consistent environmental public health indicators on lead, carbon monoxide, air, water, asthma, acute myocardial infarction, birth defects, cancer, and reproductive outcomes as well as a suite of analytical tools and services for its users. The air quality indicator is composed a group of measures including both ambient criteria air pollutants such as O_3_ and total PM_2.5_ as well as modeled air toxics. The measured air pollutant concentrations are from U.S. EPA’s Air Quality System (AQS), which covers approximately 20% of U.S. counties. In addition, most PM_2.5_ air monitors take samples every three days and many O_3_ monitors sample only during the O_3_ season. To fill the temporal and spatial data gaps, EPA developed a downscaler model to fuse daily 8-h max O_3_ concentrations and 24 h average PM_2.5_ monitoring data from the National Air Monitoring Stations/State and Local Air Monitoring Stations (NAMS/SLAMS) with 12 km gridded output from the Models-3/Community Multiscale Air Quality (CMAQ) model (Berrocal *et al.* [53]). Both AQS and downscaler datasets are available through the EPHTN to track possible exposures to O_3_ and PM_2.5_, evaluate health impact, conduct analytical studies linking health effects and the environment, and guide public health actions.

While the overwhelming majority of PM_2.5_ health effects studies worldwide use PM_2.5_ mass as the exposure metric, PM_2.5_ includes many different components, which may vary in their toxicity and in the degree to which they contribute to the mortality and other adverse health effects. To date, neither the constituents nor the emission sources that may be most associated with the increased health risks have been determined (Pope and Dockery [3]). Differential toxicity can have important implications for both the establishment of ambient air quality standards and for more targeted pollution control strategies. Specifically, focusing regulations on the most toxic PM_2.5_ constituents could protect public health at a lower total cost. A major hurdle delaying the health research and policy making on PM_2.5_ constituents is data scarcity as ground measurements at central stations can only represent average exposure of people living in their vicinity. The ARLCAS can significantly enhance Tracking’s air quality indictor by providing gridded ground-level PM_2.5_ component concentrations. Such a long-term and internally consistent dataset is not available publically elsewhere. 

CDC Tracking has the mandate to continue the collection, integration, analysis, and dissemination of environmental exposure and health data. The utility of Tracking depends on the availability, timeliness, and compatibility of existing information (Kyle *et al.* [54]; McGeehin [55]). Future development of the EPHTN will focus on expanding the Tracking website and improving data utilization by making data easier to understand (e.g., animated maps of time trends), improving quality of data (e.g., geocoding), and managing projects that link environmental and health data. The EPHTN will continue to standardize its data on the network, host data for other public health programs such as Built Environment, Climate and Health, and Developmental Disabilities, which are available to the public only through the EPHTN, and disseminate information and tools about the Network to decision makers and the public. It should be noted that although model simulated air quality information is starting to be used in public health surveillance and air pollution epidemiologic research (e.g., Brauer *et al.* [56]), it poses some challenges that must be addressed carefully. The most important one is the need to validate model estimates to ensure their accurate representation of ambient concentrations as the current air quality models still have difficulty in estimating PM_2.5_ accurately across the entire CONUS (Bell [57]). For a data assimilation system such as the NOAA ARLCAS, the availability of satellite observations to be fused into CMAQ is affected by cloud cover. The impact of the spatial heterogeneity of observations on system performance needs to be carefully evaluated as environmental surveillance requires consistent data quality in space and time.

## 5. Conclusions

This paper summarizes the progress of an ongoing effort spearheaded by the ARL, NOAA to build a prototype regional chemical analysis system (ARLCAS). The prototype focuses on providing long-term analysis surface air pollutant concentration fields over the CONUS. It leverages the expertise of a wide scope of skill and know-how from NASA’s Earth Science Division-sponsored AQAST who contributed the state-of-science knowledge in atmospheric chemical and dynamics modeling, data assimilation, derivation of dynamic LBC from the global CTM, and observation and retrieval sciences. ARLCAS complies with national center requirement protocols and aims to be maintained by a national center. It also takes advantage of the vast comprehensive land-, air- and space-based observations and high levels of sophistication in assimilation algorithms available at the national centers for both meteorology and atmospheric chemistry to assure accuracy in reconstructing the pollutant concentration fields. We develop modularized testing to investigate the efficacies of the various components of ARLCAS. To facilitate parallel tracked testing, two variant versions of ARLCAS were used in this study to test the OI data assimilation scheme. In a mimicked state implementation plan simulation for the state of Maryland, when the SIP used ARLCAS analysis field for initial and boundary condition the root mean square error was reduced by about a factor of 4 compared with one did not. We performed five sensitivity simulations with incremental increases in chemical observations and assimilation frequencies. It is shown that with the increment, the PM_2.5_ concentration in the analysis field better matches the measurements, especially for increments to include data sets based on a different platform and/or a different retrieval algorithm. Finally, a plan to apply the analysis chemical fields to an epidemiology study sponsored by the Center for Disease Control is described. Such an application signifies the versatile linkage of air quality study with human health sciences. The tangible quantification of how much the epidemiology science community can benefit from such chemical fields can be measured by testing ARLCAS’s floor-bounding case with no data assimilation, namely using the raw forecast from NOAA’s NAQFC. This experiment has been part of ARLCAS’s modularized testing.
